# Baseline 4D Flow-Derived *in vivo* Hemodynamic Parameters Stratify Descending Aortic Dissection Patients With Enlarging Aortas

**DOI:** 10.3389/fcvm.2022.905718

**Published:** 2022-06-09

**Authors:** Stanley Chu, Ozden Kilinc, Maurice Pradella, Elizabeth Weiss, Justin Baraboo, Anthony Maroun, Kelly Jarvis, Christopher K. Mehta, S. Chris Malaisrie, Andrew W. Hoel, James C. Carr, Michael Markl, Bradley D. Allen

**Affiliations:** ^1^Department of Radiology, Northwestern University, Chicago, IL, United States; ^2^Department of Biomedical Engineering, Northwestern University, Chicago, IL, United States; ^3^Department of Surgery (Cardiac Surgery), Northwestern University, Chicago, IL, United States; ^4^Department of Surgery (Vascular Surgery), Northwestern University, Chicago, IL, United States

**Keywords:** 4D flow cardiac MRI, aortic dissection (AD), cardiac MRI, type B aortic dissection (TBAD), cardiac MRI (CMR), CTA (computed tomographic angiography), MRA (magnetic resonance angiography), 4D flow

## Abstract

**Purpose:**

The purpose of our study was to assess the value of true lumen and false lumen hemodynamics compared to aortic morphological measurements for predicting adverse-aorta related outcomes (AARO) and aortic growth in patients with type B aortic dissection (TBAD).

**Materials and Methods:**

Using an IRB approved protocol, we retrospectively identified patients with descending aorta (DAo) dissection at a large tertiary center. Inclusion criteria includes known TBAD with ≥ 6 months of clinical follow-up after initial presentation for TBAD or after ascending aorta intervention for patients with repaired type A dissection with residual type B aortic dissection (rTAAD). Patients with prior descending aorta intervention were excluded. The FL and TL of each patient were manually segmented from 4D flow MRI data, and 3D parametric maps of aortic hemodynamics were generated. Groups were divided based on (1) presence vs. absence of AARO and (2) growth rate ≥ vs. < 3 mm/year. True and false lumen kinetic energy (KE), stasis, peak velocity (PV), reverse/forward flow (RF/FF), FL to TL KE ratio, as well as index aortic diameter were compared between groups using the Mann–Whitney *U* or independent *t*-test.

**Results:**

A total of *n* = 51 patients (age: 58.4 ± 15.0 years, M/F: 31/20) were included for analysis of AARO. This group contained *n* = 26 patients with TBAD and *n* = 25 patients with rTAAD. In the overall cohort, AARO patients had larger baseline diameters, lower FL-RF, FL stasis, TL-KE, TL-FF and TL-PV. Among patients with *de novo* TBAD, those with AAROs had larger baseline diameter, lower FL stasis and TL-PV. In both the overall cohort and in the subgroup of *de novo* TBAD, subjects with aortic growth ≥ 3mm/year, patients had a higher KE ratio.

**Conclusion:**

Our study suggests that 4D flow MRI is a promising tool for TBAD evaluation that can provide information beyond traditional MRA or CTA. 4D flow has the potential to become an integral aspect of TBAD work-up, as hemodynamic assessment may allow earlier identification of at-risk patients who could benefit from earlier intervention.

## Introduction

Aortic dissection (AD) is a potentially catastrophic vascular disease generally resulting from a tear in the aortic intima which results in parallel channels of blood flow known as the true lumen (TL) and false lumen (FL). Depending on subtype and extent, AD can be associated with acute hemodynamic compromise with significant morbidity and mortality rates (1). AD can be isolated to the descending aorta (Stanford type B aortic dissection/TBAD) or associated with dissections originating in the ascending aorta (Stanford type A aortic dissection/TAAD), while repaired TAAD with unresolved dissection extending into the descending aorta is classified as residual TBAD (rTAAD) (2, 3). If not fatal, all types of TBAD transition from an acute to chronic state as the aorta remodels (4). This natural history has important implications for selection and timing of treatments, with 20–50% of patients eventually requiring intervention for aneurysmal degeneration, rapid expansion, rupture, or end organ malperfusion (5–8). Current management of uncomplicated TBAD without rupture or evidence of organ ischemia includes anti-impulse therapy consisting of heart rate and blood pressure control to reduce shear stress on the aortic wall (7, 9). However, 20–50% of medically managed chronic TBAD patients eventually require surgical intervention at a certain point during their clinical course (5, 6). Therefore, routine computed tomographic angiography (CTA), or magnetic resonance angiography (MRA) is recommended for patients with chronic TBAD at 1, 3, 6, and 12 months after the dissection onset and, if stable, annually thereafter (10–13).

Currently, it is unclear which subgroups of TBAD patients are most likely to benefit from early surgical intervention such as thoracic endovascular repair (TEVAR), open elephant trunk repair (ET), or aortic graft placement (14, 15). Imaging-based risk-stratification for late complications of chronic TBAD has historically been limited to evaluation of baseline aortic diameter and growth-rate during the follow-up period (11, 13, 16). However, recent studies using 4D flow MRI have suggested that *in vivo* hemodynamic assessment of blood flow at entry tears and in the false lumen may help identify TBAD patients with growing aortas (17–20). In the current study, we seek to further expand on these findings by using 4D flow MRI to perform voxel-wise hemodynamic quantification of the TL and FL. We hypothesize that TL and FL velocity, forward and regurgitant flow, flow stasis, and kinetic energy will better correlate with adverse-aorta related outcomes (AARO) and aortic growth in patients with TBAD.

## Materials and Methods

### Study Cohort

This study was performed in accordance with two separate Institutional Review Board approved protocols. A subgroup of subjects had 4D flow MRI included as a part of their clinical imaging standard of care, were included under a retrospective IRB protocol with waiver of informed consent. A second subgroup of patients were prospectively identified and provided signed informed consent to undergo 4D flow MRI in addition to clinical standard of care imaging. All patients were recruited at a large tertiary center. Inclusion criteria includes known TBAD with ≥ 6 months of clinical follow-up after initial presentation for TBAD or after ascending aorta intervention for rTAAD patients. For the subgroup of patients included in the analysis of aortic growth-rates, ≥ 6 months of imaging follow-up was required for inclusion. Patients with prior descending aorta intervention were excluded.

### Outcomes Definitions

The electronic medical record was used to track TBAD patient outcomes. Outcomes of interest included (1) aorta-related death and (2) surgical or endovascular intervention related to TBAD in response to a large/rapidly growing aorta. The composite of these two outcomes was defined as adverse aorta related outcomes (AARO).

### Image Acquisition

All images were acquired using 1.5T MR-systems (Magneton Avanto, Aera, or Sola, Siemens Healthineers, Erlangen, Germany). Prior to 2020, subjects underwent prospective ECG gated and respiratory navigator gated 4D flow MRI covering the entire thoracic aorta in sagittal oblique orientation. For all subjects after 2020, 4D flow MRI was scanned using retrospective ECG gating. Most imaging of these newer subjects was obtained in a coronal orientation without respiratory navigator gating. 4D flow protocol changes at our institution are designed to augment clinical 4D flow implementation.

Scan parameters for prospective scans were as follows: spatial resolution = 2.5 mm^3^, field of view (FOV) = 255–365 × 340–450 mm^2^, slab thickness = 28–50 mm, temporal resolution = 36.8–65.6 msec, repetition time (TR) = 5.3–9.4 msec, echo time (TE) = 2.2–2.5 msec, flip angle = 7–15° and velocity sensitivity (venc) = 160 cm/s. Scan parameters for retrospective scans were as follows: spatial resolution = 2.5 mm^3^, FOV = 285–407 × 380–459 mm^2^, slab thickness = 25–40 mm, temporal resolution = 22.7–54.0 msec, TR = 4.9–5.8 msec, TE = 2.0–3.0 msec, flip angle = 7–15° and venc = 160 cm/s.

### Image Processing and Segmentation

**FIGURE 1:**
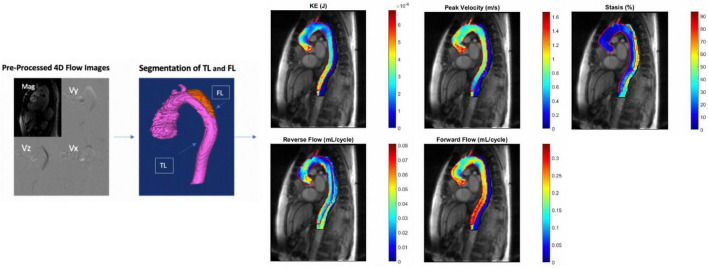
(From left to right): 4D flow MRI pre- and post-processing workflow. Eddy current correction, noise-masking of areas outside of flow regions and velocity anti-aliasing followed by manual segmentation of the aorta with TL and FL labeled. This is followed by creation of parametric hemodynamic maps. False and true lumen peak velocity, forward flow, reverse flow, kinetic energy, and stasis are each displayed on one map with TL and FL parameters overlayed. TL, true lumen; FL, false lumen; KE, kinetic energy.

A schematic of the image pre-and post-processing workflow is shown in Figure 1. Pre-processing of the 4D flow MRI data included eddy current correction, noise-masking of areas outside of flow regions and velocity anti-aliasing (MatLab; MathWorks, Natick, MA, United States) (21, 22). Time-averaged magnitude and time-averaged 3D phase-contrast angiogram (PC-MRA) images were generated to show vessel anatomy. Time-averaged magnitude images were used to manually segment the entire aorta (TL + FL) excluding aortic arch branch vessels from the aortic valve down to the first branch of the celiac artery by one of two independent observers on a designated software (Mimics Innovation Suite; Materialise, Leuven, Belgium). Subsequently, the TL was manually segmented based on PC-MRA images. The FL segmentations were determined by subtracting the TL segmentation from the whole aorta segmentation.

### Parametric Hemodynamic Maps

3D parametric maps of aortic hemodynamics were generated using in house analysis tools (MatLab; MathWorks, Natick, MA, United States) similar to a recently reported workflow (23). The 4D flow velocity data were interpolated to 1 mm^3^ using spline interpolation. For each voxel inside both the TL and the FL, kinetic energy (KE), forward flow (FF), reverse flow (RF), stasis, and peak velocity (PV) were calculated.

Because our study cohort included both retrospectively and prospectively-gated scans, the minimum % cardiac cycle imaged in any single prospective scan (62.9%) was used as a cutoff for the entire cohort when creating parametric hemodynamic maps and calculating aortic voxel-wise and volumetric sums. These percentages were calculated using each patients’ respective heart rate during their baseline 4D flow scan.

#### Forward Flow and Reverse Flow

A 3D aortic centerline was automatically calculated using the TL segmentation and orthogonal analysis planes were automatically placed every millimeter along the center line. The plane served to determine the direction of the forward flow from the normal vector. Each voxel was matched to the nearest plane to determine the forward or reverse flow for each time point. FF. Reported FF and RF is calculated by first summing each voxel over the cardiac cycle, and then averaging these sums over an entire luminal volume.

#### Kinetic Energy

Voxel-wise KE was determined by:


(1) $$•\mathrm{KE}=0.5\times \rho \times \text{dV}\times \text{v}{\left(\text{t}\right)}^{2}$$

with ρ the blood density assumed as 1060 kg/m^3^ and dV the unit voxel volume (i.e., 1 mm^3^). Reported KE is calculated by first summing each voxel over the cardiac cycle, and then averaging these sums over an entire luminal volume.

#### Kinetic Energy Ratio

FL KE divided by TL KE.

#### Peak Velocity

The time point with the maximum 95th percentile voxel-wise peak velocity was used to determine the 3D peak velocity maps. The time points were selected independently for TL and FL. The average of the maximum top 5% velocities is reported as peak velocity for both lumens.

#### Stasis

Voxel-wise flow stasis was defined as the percentage of the cardiac cycle that the velocity in that voxel is < 0.1 m/s –this definition was used for generation of stasis maps. Reported stasis was calculated by averaging these percentages over an entire luminal volume.

All hemodynamic parameters were then indexed to maximum aortic size (the measurement process is detailed below) by dividing each patient’s parameters by their respective baseline aortic diameter, and are reported as such.

### Morphologic Measures and Aorta Growth Rate

For each subject, standard high resolution MRA images were also included as a part of the MR protocol. Maximal dissection diameter (“baseline diameter”), (including both true and false lumens), entry tear diameter, and false lumen maximal diameter were obtained using dedicated visualization and multiplanar reformation software (Visage 7, Visage Imaging, Inc., San Diego, CA, United States). To be included in the growth rate sub analysis, aortic diameters were measured from aortic CTA or MRA imaging acquired with at least 180-day interval between scans. All morphologic measurements were performed by the same experienced cardiovascular radiologist (BDA).

### Interobserver Study

Two independent, double-blinded observers completed the pre-processing and manual segmentations of the 4D flow MRI data separately in a subgroup of subjects representative of 22% of the total cohort.

### Statistical Analysis

A Shapiro-Wilk normality test was used to assess the distribution of the data. The patient cohort was divided into 2 groupings based on: (1) rapid vs. slow aortic growth (defined as ≥ 3 mm/year vs. < 3 mm/year) and (2) presence vs. absence of interval AARO. This threshold for rapid aortic growth is based on studies that found “high risk” dissection patients had a mean aortic growth rate of ∼3mm/year, and has been used as a definition in other similar studies (18, 24, 25). For all groupwise comparisons, an independent T test was used for normally distributed data, while a Mann–Whitney *U* test was used for non-normally distributed parameters. Analysis of AARO included the full patient cohort with ≥ 6 months of clinical follow-up. To control for significant univariate predictors, multivariable binary logistic regression was performed using conditional backward stepwise removal of variables that were significant on univariate analysis. This analysis was not performed in subgroup analysis due to limited sample size of the *de novo* TBAD and rTAAD subgroups.

Comparisons of rapid vs. slow aortic growers only included the subgroup of patients with ≥ 6 months of imaging follow-up. A Pearson correlation test was also used to assess correlations between hemodynamic parameters and aortic growth rate.

Chi-squared analysis was use for comparisons of categorical demographic parameters between groups. Bland-Altman analysis, correlations, and Sørensen-Dice analysis were performed for the interobserver comparisons. Statistical significance level was set to alpha = 0.05. *Post-hoc* power analysis was performed for baseline diameter differences between AARO and non-AARO patients in the overall cohort, to make sure the study is powered to detect differences of this reference standard measurement. All statistical tests above were performed in SPSS 20.0 (International Business Machines Corporation, United States).

## Results

### Patient Demographics

**FIGURE 2:**
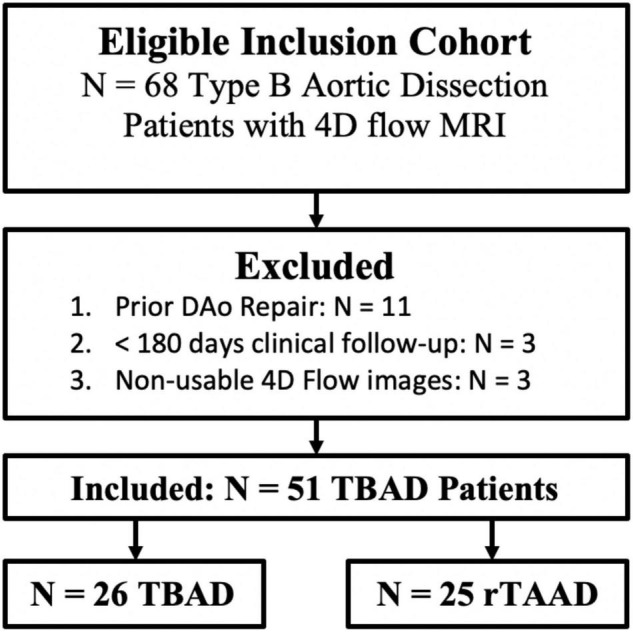
Cohort development flow-chart. TBAD, Type B Aortic Dissection; rTAAD, repaired type A aortic dissection.

A total of *n* = 68 patients were eligible for inclusion, with patients excluded due to prior descending aorta intervention (*n* = 11), < 180 days of clinical follow-up (*n* = 3), or non-usable 4D flow data (*n* = 3). Therefore, *n* = 51 patients (58.4 ± 15.0 years, 31 (61%) male) were included in the final cohort (Figure 2). Of these scans, *n* = 28 (55%) were retrospectively-gated, while *n* = 23 (45%) were prospectively-gated. This group includes *n* = 25 (49%) rTAAD, and *n* = 26 (51%) TBAD cases. A total of *n* = 12 (24%) patients had TBAD-related adverse outcomes, including aorta-related death (*n* = 2), TEVAR (*n* = 6), elephant trunk repair (*n* = 2) or descending aorta graft repair (*n* = 1). For the entire cohort, the clinical follow-up interval since presentation is 4.9 ± 5.1 years, the time from presentation to 4D flow is 2.8 ± 3.9 years, and the follow-up interval after 4D flow is 2.2 ± 2.5 years.

Within this cohort of 51 patients, *n* = 9 patients had < 180 days of imaging follow-up. During statistical analysis regarding aortic growth rate, these patients were excluded, leaving *n* = 42. Demographic and follow-up information further broken down by aortic growth groups are detailed in Table 1.

**TABLE 1:** Comparison of demographic factors in the study cohort, divided into (1) adverse outcome vs. no outcome groups and (2) aortic growth ≥ 3 mm/year vs. aortic growth < 3 mm/year.

Demographic			Scans with > 180d clinical F/U (*n* = 51)			Scans with > 180d imaging F/U (*n* = 42)		
		
			Adverse outcome (*n* = 12)	No outcome (*n* = 39)	*P*-value	Growth ≥ 3 mm/year (*n* = 10)	Growth < 3 mm/year (*n* = 32)	*P*-Value
Age (years)		Mean	55.97 ± 8.87	60.22 ± 13.46	0.21	51.33 ± 11.59	61.52 ± 13.16	0.03*
BMI			25.77 ± 6.60	29.69 ± 8.32	0.19	27.56 ± 7.80	29.53 ± 8.52	0.50
Systolic blood pressure (mmHg)			129.83 ± 25.25	125.96 ± 16.65	0.67	128.30 ± 13.14	125.48 ± 18.98	0.96
Pulse pressure (mmHg)			55.25 ± 15.23	52.42 ± 13.33	0.57	51.32 ± 19.91	52.94 ± 11.80	0.73
Heart rate (bpm)			75.75 ± 13.65	70.94 ± 13.72	0.30	74.80 ± 15.07	70.72 ± 12.11	0.45
Mean clinical follow-up time since 4D flow MRI (Years)			7.15 ± 8.61	4.04 ± 3.48	0.83	N/A	N/A	N/A
Mean imaging follow-up time (Years)			N/A	N/A	N/A	1.23 ± 0.73	3.17 ± 2.34	0.02*

Male gender		*n* (%)	9(75)	23(59)	0.50	7(70)	19(59)	0.72
Acuity	Acute		3(25)	6(15)	0.67	1(10)	3(9)	1.00
	Subacute		2(17)	2(5)	0.23	0(0)	2(6)	1.00
	Chronic		7(58)	31(79)	0.25	8(80)	27(84)	1.00
Prior type A repair			3(25)	22(56)	0.10	5(50)	19(59)	0.72
Prior Aao/AV surgery			0(0)	4(10)	0.56	1(10)	2(6)	1.00
Positive smoking history			7(58)	20(51)	0.67	6(60)	14(44)	0.37
Medications	Anti-hypertensive		10(83)	39(100)	0.05	9(90)	31(97)	0.42
	Aspirin		7(58)	27(69)	0.73	6(60)	21(54)	1.00
	Statin		7(58)	28(72)	0.48	6(60)	22(56)	0.71
	Warfarin		2(17)	1(3)	0.13	2(20)	1(3)	0.14

No demographic variables significantly differed in any group comparisons, except the no outcome group had more patients with prior type A repair than the adverse outcome group (p = 0.03). *P < 0.05. F/U = follow-up.

*BMI = body mass index. Aao = ascending aorta. AV = aortic valve. Bold indicates significance.*

Demographic comparisons between study groups are also provided in Table 1. No demographic factors significantly differed between patients with/without adverse outcomes (*p* < 0.05). Patients with slow aortic growth were older (61.52 ± 13.16 vs.51.33 ± 11.59, *p* = 0.03) and had higher imaging follow-up time (3.17 ± 2.34 vs. 1.23 ± 0.73 years, *p* = 0.02). There were no other demographic factors that differed between patients with aortic growth groups (*p* > 0.05).

### Overall Cohort

#### Adverse-Aorta Related Outcomes

**FIGURE 3:**
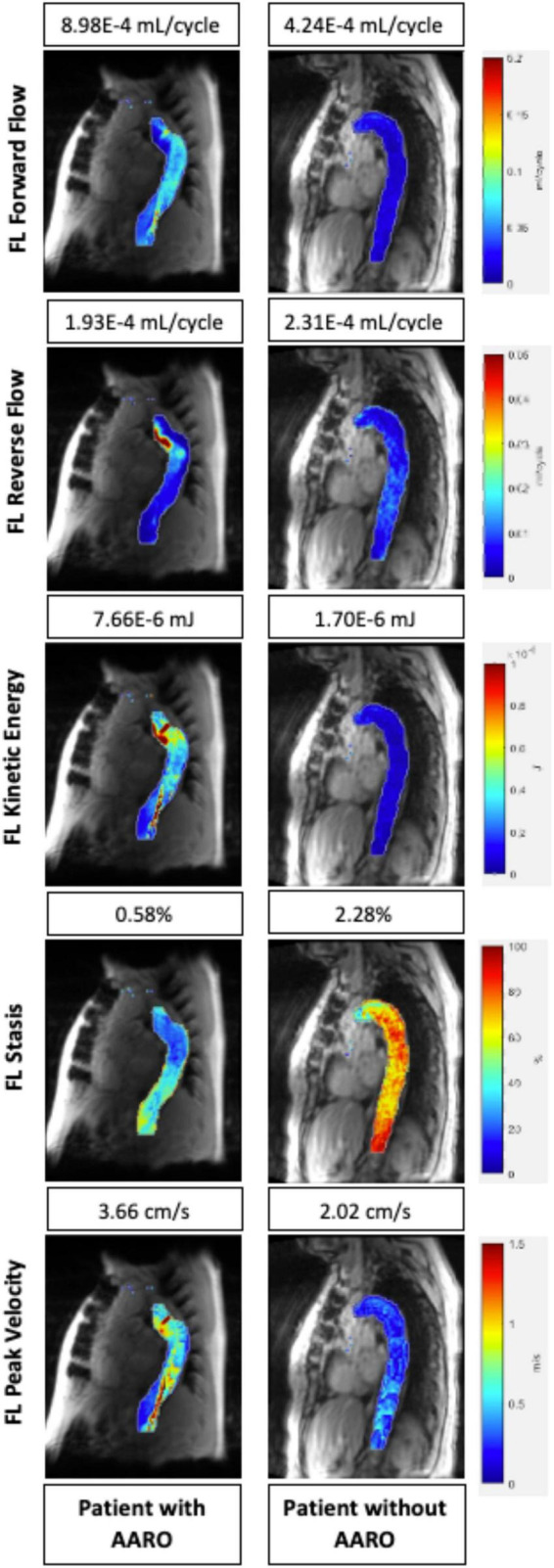
Parametric hemodynamic maps comparing FL forward flow, reverse flow, kinetic energy, stasis, and peak velocity in two patients (one with AARO, the other without AARO). The magnitude of each parameter for both patients are also listed above the parametric map (numbers from top to bottom: forward flow, reverse flow, kinetic energy, stasis, and peak velocity. FL, false lumen; AARO, adverse aorta related outcome.

Table 2 a total of *n* = 12 patients (24%) had adverse outcomes. The average time to event was: 233 ± 252 days (range: [1, 720]). On univariate analysis, AARO patients had larger baseline aortic diameters (51 ± 7 mm vs. 42 ± 7 mm, *p* = 0.001), entry tear diameter (11 ± 4 mm vs. 8 ± 8 mm, *p* = 0.004), and FL diameter (46 ± 7 mm vs. 38 ± 9 mm, *p* = 0.003). However, they had lower FL reverse flow (2.21E-4 ± 9.44E-5 vs. 3.13E-4 mL/cycle ± 1.46E-4 mL/cycle, *p* = 0.02), FL stasis (1.16 ± 0.36% vs. 1.55 ± 0.46%, *p* = 0.01), TL kinetic energy (1.86E-5 ± 1.03E-5 mJ vs. 2.82E-5 ± 1.38E-5 mJ, *p* = 0.02), and TL forward flow (2.04E-3 ± 7.09E-4 mL/cycle vs. 2.76E-3 ± 9.57E-5 mL/cycle, *p* = 0.01), and TL peak velocity (3.84 ± 1.37 cm/s vs. 5.70 ± 2.05 cm/s, *p* = 0.01). A representative example of hemodynamic parametric maps in a patient with AARO compared to a patient without is provided in Figure 3.

**TABLE 2:** Hemodynamic and morphologic comparisons between (1) patients with and without adverse outcomes (2) rapid v. slow aortic growth.

Overall cohort							

Indexed parameter (per mm)	No adverse outcome (*n* = 39)	Adverse outcome (*n* = 12)	*P*-value	*P*-value (MV)	Rapid aortic growth (*n* = 10)	Slow aortic growth (*n* = 32)	*P*-value
FL mean reverse flow (mL/cycle)	3.13E-4 ± 1.46E-4	2.21E-4 ± 9.44E-5	0.02*	–	2.37E-4 ± 1.36E-4	2.98E-4 ± 1.46E-4	0.09
FL mean stasis (%)	1.55 ± 0.46	1.16 ± 0.36	0.01*	0.046*	1.39 ± 0.36	1.46 ± 0.46	0.59
TL mean KE (mJ)	2.82E-5 ± 1.38E-5	1.86E-5 ± 1.03E-5	0.02*	–	2.29E-5 ± 1.06E-5	2.75E-5 ± 1.48E-5	0.46
TL mean forward flow (mL/cycle)	2.76E-3 ± 9.57E-4	2.04E-3 ± 7.09E-4	0.01*	–	2.29E-3 ± 8.61E-4	2.63E-3 ± 8.68E-4	0.43
TL peak velocity (cm/s)	5.70 ± 2.05	3.84 ± 1.37	0.01*	0.03*	4.75 ± 1.61	5.45 ± 2.22	0.61
Baseline diameter (mm)	41.97 ± 7.33	50.50 ± 6.87	0.001*	–	45.90 ± 6.12	43.25 ± 8.00	0.28
Entry tear diameter (mm)	7.59 ± 8.25	11.00 ± 3.69	0.004*	–	8.50 ± 4.99	7.13 ± 6.94	0.32
FL diameter (mm)	38.33 ± 8.50	46.17 ± 6.77	0.003*	–	41.50 ± 4.93	39.56 ± 7.81	0.47
KER	3.11E-3 ± 2.34E-3	3.93E-3 ± 2.85E-3	0.41	–	4.23E-3 ± 2.04E-3	3.02E-3 ± 2.34E-3	0.03*

These groupwise comparisons are explored in the overall cohort. Statistically significant differences are highlighted in bold. *P < 0.05. P-value (MV) = p-value derived from multivariate binary logistic regression.

When controlling for other significant univariate predictors with binary logistic regression, only FL stasis (*p* = 0.046) and TL peak velocity (*p* = 0.03) remained significantly different between AARO and non-AARO patients.

*Post-hoc* power analysis found that the study had a statistical power of 0.96 to detect the observed difference in baseline diameter between AARO and non-AARO patients in the overall cohort. Results of power analysis of all significant hemodynamic and morphologic parameters in the overall cohort are detailed in Supplementary Table 1.

### *De novo* Type B Aortic Dissection

#### Adverse-Aorta Related Outcomes

A total of *n* = 9 patients (35%) had adverse outcomes. The average time to event was: 160 ± 233 days (range: [1, 720]). On univariate analysis, AARO patients had larger baseline aortic diameter (51 ± 7 mm vs. 42 ± 9 mm, *p* = 0.02) and FL diameter (47 ± 8 mm vs. 37 ± 10 mm, *p* = 0.03). However, they had lower FL stasis (1.16 ± 0.40% vs. 1.49 ± 0.51%, *p* = 0.03) and TL peak velocity (3.68 ± 1.33 cm/s vs. 5.78 ± 2.52 cm/s, *p* = 0.03). Forward flow, reverse flow and kinetic energy did not significantly differ between groups (Table 3).

**TABLE 3:** Subgroup analysis of hemodynamic and morphologic comparisons between (1) patients with and without adverse outcomes (2) rapid v. slow aortic growth.

*De novo TBAD*									

Indexed parameter (per mm)		No adverse outcome (*n* = 17)	Adverse outcome (*n* = 9)	*P*-value	*P*-value (MV)	Rapid aortic growth (*n* = 5)	Slow aortic growth (*n* = 13)	*P*-value	*P*-value (MV)
FL mean stasis (%)		1.59 ± 0.51	1.16 ± 0.40	0.03*	0.15	1.28 ± 0.22	1.51 ± 0.58	0.24	–
FL mean reverse flow (mL/cycle)		2.82E-4 ± 1.09E-4	2.39E-4 ± 1.01E-4	0.31	–	1.80E-4 ± 4.99E-5	2.55E-4 ± 8.41E-5	0.04*	0.99
TL peak velocity (cm/s)		5.78 ± 2.52	3.68 ± 1.33	0.03*	0.13	3.45 ± 9.78E-1	5.59 ± 2.72	0.21	–
KER		2.53E-3 ± 1.57E-3	3.95E-3 ± 3.23E-3	0.39	–	4.85E-3 ± 1.56E-3	2.11E-3 ± 1.07E-3	0.01*	0.99
Baseline diameter (mm)		41.59 ± 9.02	50.67 ± 7.94	0.02*	–	46.20 ± 7.60	44.23 ± 10.49	0.67	–
FL diameter (mm)		37.41 ± 10.40	46.56 ± 7.84	0.03*	–	41.40 ± 3.78	40.15 ± 9.37	0.48	–

	**Repaired TAAD with Residual TBAD**								

**Indexed parameter (per mm)**		**No adverse outcome (*n* = 22)**	**Adverse outcome (*n* = 3)**	–	–	**Rapid aortic growth (*n* = 5)**	**Slow aortic growth (*n* = 19)**	***P*-value**	***P*-value (MV)**

FL mean reverse flow (mL/cycle)		3.38E-4 ± 1.67E-4	1.77E-4 [1.15E-4, 2.10 E-4]	–	–	2.93E-4 ± 1.77E-4	3.28E-4 ± 1.72E-4	0.68	–
TL mean FF (mL/cycle)		2.79E-3 ± 8.78E-4	1.90E-3 [1.38E-3, 1.94E-3]	–	–	2.69E-3 ± 9.81E-4	2.65E-3 ± 9.24E-4	0.68	–
Baseline diameter (mm)		41.77 ± 5.83	51 [47, 52]	–	–	45.60 ± 5.13	42.58 ± 5.98	0.30	–
Entry Tear diameter (mm)		5.73 ± 4.93	9 [9, 10]	–	–	5.60 ± 3.65	6.84 ± 5.00	0.55	–
FL diameter (mm)		39.05 ± 6.87	44 [44, 47]	**–**	–	41.60 ± 6.35	39.16 ± 6.78	0.48	–

*Note that statistical comparison for AARO was not performed for the rTAAD with residual TBAD subgroup due to small number of subjects. Statistically significant differences are highlighted in bold.*

*P < 0.05. TBAD, type B aortic dissection; TAAD, type A aortic dissection. Data for the rTAAD with AARO patient group is presented as median [min, max].

Statistical comparison was not performed for AARO in rTAAD due to the small number of subjects (*n* = 3) of rTAAD patients with AARO (Table 3).

### Aortic Growth Rate

A total of *n* = 42 patients had at least 180 days of imaging follow-up including *n* = 18 *de novo* TBAD (69% of subgroup) and *n* = 24 rTAAD (96 of subgroup). The average follow-up imaging interval was 2.7 ± 2.2 years.

For *de novo* TBAD, the mean diameter change was 6 ± 5 mm with a growth rate of 3.2 ± 4.8 mm/yr. In rTAAD, the mean diameter change was 2 ± 4 mm with a growth rate of 1.5 ± 4.1 mm/yr. There was no significant difference in growth rate of AARO and non-AARO groups for either TBAD or rTAAD.

In the overall cohort, patients with rapid aortic growth had higher kinetic energy ratio between the FL and TL (4.23E-3 ± 2.04E-3 vs. 3.02E-3 ± 2.34E-3, *p* = 0.03; Table 2). No other significant correlations or groupwise differences were present for hemodynamic or morphologic parameters in the overall cohort.

**FIGURE 4:**
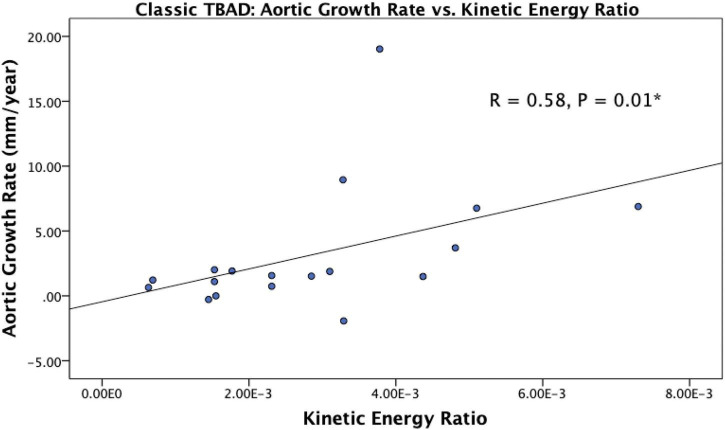
Scatter plot showing the relationship between kinetic energy ratio and aortic growth rate in *de novo* TBAD patients. Spearman correlation coefficient is reported as R. Kinetic energy ratio was positively correlated with aortic growth rate (*r* = 0.58, *p* = 0.01). *P < 0.05.

*De novo* TBAD patients with rapid aortic growth had a lower FL reverse flow (1.80E-4 ± 4.99E-5 mL/cycle vs. 2.55E-4 ± 8.41E-5 mL/cycle, *p* = 0.04) and a higher kinetic energy ratio (4.85E-3 ± 1.56E-3 vs. 2.11E-3 ± 1.07E-3, *p* = 0.01; Table 2). Kinetic energy ratio was positively correlated with aortic growth rate (*r* = 0.58, *p* = 0.01) in *de novo* TBAD patients (Figure 4). No significant correlations or groupwise differences were present for hemodynamic or morphologic parameters in the rTAAD cohort.

### Interobserver Study

The results for DICE-scores, correlations and Bland-Altman analysis are shown in Supplementary Table 2 and Supplementary Figure 1. The interobserver study included 11 subjects from the overall cohort of 51 (4 with rapid aortic growth, 1 with AARO - death). Intraclass correlation coefficients (ICC) in the TL were: KE (0.97, *p* < 0.05), MV (0.99, *p* < 0.05), stasis (0.98, *p* < 0.05), FF (0.98, *p* < 0.05) and RF (0.96, *p* < 0.05). ICCs in the FL were: KE (0.97, *p* < 0.05), MV (0.99, *p* < 0.05), stasis (0.99, *p* < 0.05), FF (0.99, *p* < 0.05), RF (0.98, *p* < 0.05). DICE coefficients for the TL and FL were 0.87 [95% CI: 0.84-0.91] and 0.81 [95% CI: 0.79-0.88], respectively.

## Discussion

In our study of 4D flow-derived hemodynamics in a heterogenous cohort including both *de novo* TBAD and rTAAD patients, there were several interesting results. First, multiple hemodynamic parameters in both TL and FL were different between patients with/without AARO in the combined cohort of *de novo* TBAD and rTAAD. Second, these hemodynamic parameters were unique subgroups. *De novo* TBAD patients with AARO had lower FL stasis and TL peak velocity. Finally, aortic diameter did not differ between rapid and slow growers, but *de novo* TBAD patients with rapid aortic growth had lower FL reverse flow and a higher kinetic energy ratio.

Previous studies have identified potential use for 4D flow MRI in assessment of aortic growth after TBAD. Recently, Marlevi et al. found that TBAD patients with enlarging aortas (≥3 mm/year) had higher FL ejection fraction and FL pressure in a cohort of 12 patients with chronic TBAD (18). Our study expands on existing work by investigating the role of 6 additional hemodynamic parameters within a larger cohort of 51 patients. One unique feature of our study is unique due to the inclusion of patients with both *de novo* TBAD as well as rTAAD with residual TBAD. There has been growing evidence that these two patient populations differ hemodynamically, with Jarvis et al. finding significantly altered regional differences in both TL and FL flow parameters between *de novo* TBAD and rTAAD patients (23). Our results expanded on this idea, as we found additional hemodynamic parameters that are predictive of AARO and/or rapid aortic growth differed amongst these patient populations. While our small sample size (*n* = 3 rTAAD patients with adverse outcomes, *n* = 5 rTAAD patients with rapid aortic growth) limits our ability to statistically analyze these subgroups, the trends are compelling, and further studies with larger sample sizes are necessary. Nonetheless, our results continue to suggest that *de novo* TBAD and rTAAD patients should not be risk-assessed in the same way. Another unique feature of this study is our analysis of adverse aorta related outcomes. Previous studies have investigated aortic growth rate, which is what current TBAD risk assessment centers around, but our results show that a single baseline hemodynamic assessment of TL and FL flow parameters may identify physiologic drivers increasing the risk of eventual AARO before the 3, 6, or 12 months of serial imaging needed to calculate growth-rate (10, 12, 26).

Other aspects of our results are also compelling in the context of previous TBAD studies. In particular, our finding that *de novo* TBAD patients without AARO had higher FL stasis suggests that sluggish flow in the FL is protective. This hypothesis is in line with the mechanism of action of TBAD treatment strategies such as TEVAR and anti-impulse therapy, and supports evidence that FL thrombus is protective (11). Hemodynamics in the true lumen may also signify elevated AARO risk. For example, *de novo* TBAD patients with AARO had lower TL peak velocity, while rTAAD patients with AARO had lower TL forward flow. This result is interesting in the context of a 2018 study by Liu et al., where false lumen entry tear size was negatively correlated with TL flow velocity (*p* < 0.05) (27). Thus, our findings could be explained by assuming that a larger entry tear would result in higher flow into the false lumen, thereby diverting true lumen flow. This assumption is supported by the finding that AARO patients in the overall cohort had significantly larger entry tear and FL diameters (*p* < 0.05). In other words, decreased TL flow volume/velocity could be an indirect marker of increased FL flow, potentially contributing to vessel damage. Importantly, on multivariate modeling, we found that hemodynamic parameters remained significant predictors of AARO even when controlling for aortic, false lumen, and entry tear diameters.

Finally, baseline aortic diameter—traditionally used to surveil TBAD progression—was different between AARO and non-AARO groups. This is not surprising given that current management paradigms strongly consider aortic diameter in the treatment algorithm of TBAD patients. Of note, baseline diameter was not predictive of rapid growth, nor was it significantly correlated with aortic growth rate. However, we found that patients with rapid aortic growth in both the overall and *de novo* TBAD cohorts had significantly higher kinetic energy ratio, and KER was positively correlated with aortic growth rate in *de novo* TBAD patients as well. This parameter potentially highlights differences in dynamic pressure between the TL and FL as a driver for rapid aortic growth. Considering the larger ET and FL diameters seen with AARO patients, we can speculate that this dynamic pressure difference may stem from larger FL filling in AARO patients. Moreover, *de novo* TBAD patients with rapid aortic growth had lower FL reverse flow. Again, retrograde flow in the FL (specifically at the dissection tear) was analyzed as FL ejection fraction (EF: diastolic retrograde flow divided by systolic antegrade flow through an entry tear) in 18 patients by Burris et al. and in 12 patients by Marlevi et al. Both studies reported a strong positive correlation between FL EF and aortic growth rate, contrary to the negative correlation that we found (17, 18). This discrepancy may due to the fact that our analysis of reverse flow involves data collected from an entire luminal volume, and further studies focusing on a specific location of interest may reveal regional hemodynamic differences in this parameter. In addition, our small number of rapid growers (*n* = 10) may have limited our ability to detect further hemodynamic differences. Nonetheless, our results suggest that alternative quantitative measures besides aortic diameter are clearly needed to risk-stratify TBAD patients in terms of aortic growth, and our study identifies FL reverse flow and kinetic energy ratio as potential targets.

This study has several limitations. First, the observational nature of the study may have biased our evaluation of hemodynamic and morphologic parameters, especially because decisions to surgically intervene in our cohort were likely influenced by aortic size. Randomized controlled trials would be needed to better evaluate these metrics. Second, technical imaging development at our institution led to a transition from prospectively to retrospectively ECG-gated 4D flow MRI, with both scan types included in our study. This may have affected our ability to detect further differences in hemodynamic parameters, particularly during late diastole. Considering the low velocity and blood stasis expected in the FL, the low velocity to noise ratio (venc = 160 cm/s) in our imaging may have also impacted our analysis of FL hemodynamics, particularly regarding flow stasis. Multi-venc or dual-venc 4D Flow MRI acquisitions using low and high-venc data together may help to address this issue in future studies (28, 29). Low velocity to noise ratio due to VENC may impact measurements, dual venc could help address the problem. Last, lumen segmentation was performed by two independent observers, increasing the risk of bias during image post-processing. While there was mild variation between segmenters in the interobserver analysis, the high DICE scores and interclass correlation coefficients > 0.95 indicate good agreement.

## Conclusion

Our study suggests that 4D flow MRI is a promising tool for TBAD evaluation that can provide information beyond traditional MRA or CTA. 4D flow has the potential to become an integral aspect of TBAD work-up, as hemodynamic assessment may allow earlier identification of at-risk patients who could benefit from earlier intervention.

## Data Availability Statement

The original contributions presented in the study are included in the article/Supplementary Material, further inquiries can be directed to the corresponding author.

## Author Contributions

SC: lumen segmentation, data processing, statistical analysis, and manuscript writing. OK: segmentation, manuscript, and editing. MP: segmentation guidance and manuscript editing. EW: data processing, manuscript, and editing. JB: data retrieval and manuscript editing. AM, KJ, CM, SM, AH, and JC: manuscript editing. BA and MM: manuscript editing and statistical analysis. All authors contributed to the article and approved the submitted version.

## Conflict of Interest

The authors declare that the research was conducted in the absence of any commercial or financial relationships that could be construed as a potential conflict of interest.

## Publisher’s Note

All claims expressed in this article are solely those of the authors and do not necessarily represent those of their affiliated organizations, or those of the publisher, the editors and the reviewers. Any product that may be evaluated in this article, or claim that may be made by its manufacturer, is not guaranteed or endorsed by the publisher.

## Abbreviations

TBAD: type B aortic dissection
TAAD: type A aortic dissection
rTAAD: repaired TAAD with residual TBAD
TEVAR: thoracic endovascular aortic repair
CTA: computed tomography angiography
MRA: magnetic resonance angiography
PC-MRA: 3D phase-contrast angiogram
AARO: adverse aorta-related outcomes
FOV: field of view
TR: repetition time
TE: echo time
VENC: velocity encoding
TL: true lumen
FL: false lumen
KE: kinetic energy
PV: peak velocity
FF: forward flow
RF: reverse flow.
