# Supplementary material for: Baseline 4D Flow-Derived in vivo Hemodynamic Parameters Stratify Descending Aortic Dissection Patients With Enlarging Aortas
Source: Front Cardiovasc Med. 2022 Jun 9;9:905718. doi: 10.3389/fcvm.2022.905718 (PMC9218246; doi:10.3389/fcvm.2022.905718)

**Supplemental Table 1:** Results of post-hoc power analysis for hemodynamic and morphologic parameters that significantly differed between various groups in the overall cohort. AARO: adverse aorta-related event. FL: false lumen. TL: true lumen. KE: kinetic energy. KER: kinetic energy ratio.

| **Overall Cohort** | | |
| --- | --- | --- |
| **Indexed Parameter** | **Post-Hoc Power** | |
|  | **No AARO (n = 39) vs. AARO (n = 12)** | **Rapid Aortic Growth (n = 10) vs. Slow Aortic Growth (n = 32)** |
| FL Mean Reverse Flow | 0.58 | 0.21 |
| FL Mean Stasis | 0.80 | 0.08 |
| TL Mean KE | 0.63 | 0.16 |
| TL Mean Forward Flow | 0.70 | 0.18 |
| TL Peak Velocity | 0.87 | 0.16 |
| Baseline Diameter | 0.95 | 0.17 |
| Entry Tear Diameter | 0.34 | 0.09 |
| FL Diameter | 0.84 | 0.13 |
| KER | 0.15 | 0.31 |

**Supplemental Table 2: A)** Median Dice scores comparing the true lumen and false lumen segmentations between two independent observers. B & C) Bland-Altman statistics comparing hemodynamic parameters (KE, PV, stasis, FF, and RF) in the TL and FL, respectively. These parameters were calculated from segmentations performed by two independent observers. There was no significant bias in either the TL or FL parameters (p < 0.05).

1. **Dice scores (DSC)**

TL median DSC: 0.87 [0.84 - 0.91]

FL median DSC: 0.81 [0.79 - 0.88]

| **Bland-altman**  **statistic** | **TL Hemodynamic parameters (*n =* 10)** | | | | |
| --- | --- | --- | --- | --- | --- |
|  | **KE (**$\boldsymbol{\mu J}$**)** | **Peak Velocity (m/s)** | **Stasis (%)** | **Forward Flow (mL/cycle)** | **Reverse Flow (mL/cycle)** |
| Bias | -0.01 | 0.01 | -1.14 | -0.01 | 0.00 |
| SD of Bias | 0.02 | 0.08 | 2.41 | 0.01 | 0.01 |
| Min., Max. Limit (95%) | [-0.05. 0.03] | [-0.15, 0.17] | [-5.87, 3.58] | [-0.02, 0.02] | [-0.01, 0.01] |
| *p-*value | 0.31 | 0.80 | 0.71 | 0.55 | 0.97 |
|  |  |  |  |  |  |
|  |  |  |  |  |  |

| **Bland-altman**  **statistic** | **FL Hemodynamic parameters (*n =* 10)** | | | | |
| --- | --- | --- | --- | --- | --- |
|  | **KE (**$\boldsymbol{\mu J}$**)** | **Peak Velocity (m/s)** | **Stasis (%)** | **Forward Flow (mL/cycle)** | **Reverse Flow (mL/cycle)** |
| Bias | 0.01 | 0.01 | -0.23 | 0.01 | -0.01 |
| SD of Bias | 0.01 | 0.03 | 3.27 | 0.01 | 0.01 |
| Min., Max. Limit (95%) | [-0.01, 0.01] | [-0.04, 0.06] | [-6.63, 6.18] | [-0.01, 0.01] | [-0.01, 0.01] |
| *p-*value | 0.77 | 0.24 | 0.83 | 0.25 | 0.67 |

|  |  |  |  |  |  |
| --- | --- | --- | --- | --- | --- |

**Supplemental Fig. 1:** Bland-Altman analysis for interobserver comparison between the two independent observers involved in manual segmentation of the TL/FL in this study. Limits of agreement for the TL are shown with the dotted blue line. Limits of agreement for the FL are shown with the dotted black line.


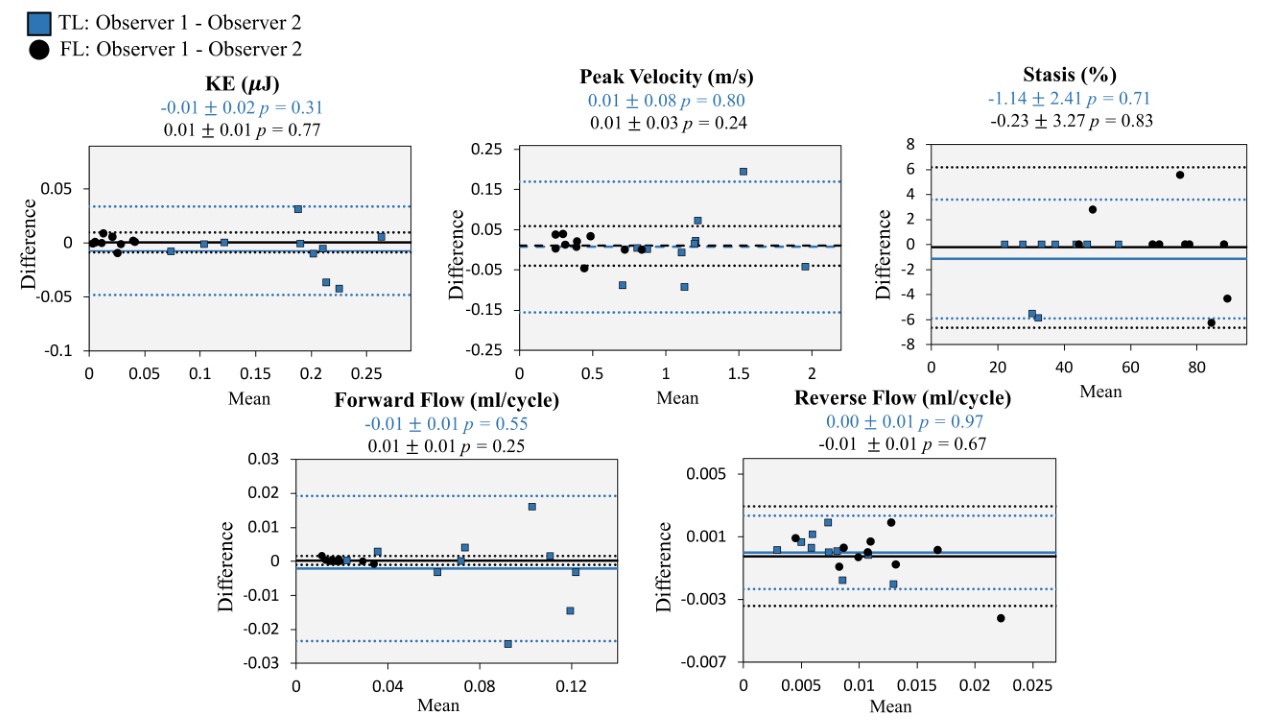

Supplement: Supplementary file 1 [file Data_Sheet_1.docx]
